# Recent Advances in the Discovery of Haem-Targeting Drugs for Malaria and Schistosomiasis

**DOI:** 10.3390/molecules14082868

**Published:** 2009-08-04

**Authors:** Katherine A. de Villiers, Timothy J. Egan

**Affiliations:** 1 University of Stellenbosch, Private Bag X1, Matieland 7602, South Africa; 2 University of Cape Town, Private Bag, Rondebosch 7701, South Africa; E-mail: timothy.egan@uct.ac.za (T.J.E.)

**Keywords:** haem, haemozoin, high-throughput screening, rational drug design

## Abstract

Haem is believed to be the target of some of the historically most important antimalarial drugs, most notably chloroquine. This target is almost ideal as haem is host-derived and the process targeted, haemozoin formation, is a physico-chemical process with no equivalent in the host. The result is that the target remains viable despite resistance to current drugs, which arises from mutations in parasite membrane transport proteins. Recent advances in high-throughput screening methods, together with a better understanding of the interaction of existing drugs with this target, have created new prospects for discovering novel haem-targeting chemotypes and for target-based structural design of new drugs. Finally, the discovery that *Schistosoma mansoni* also produces haemozoin suggests that new drugs of this type may be chemotherapeutic not only for malaria, but also for schistosomiasis. These recent developments in the literature are reviewed.

## Introduction

During development inside the red blood cell, the malaria parasite *P. falciparum* digests between 60 and 80% [1] of the available haemoglobin in an acidic food vacuole [2,3]. As a consequence of the proteolytic breakdown of haemoglobin, iron (II) protoporphyrin IX (Fe(II)PPIX) is released into an aqueous environment. The metal centre is irreversibly oxidised to Fe(III), presumably by molecular oxygen, although the detailed mechanism is not yet known. The resultant HO-/H_2_O-Fe(III)PPIX (hydroxo-/aqua-ferriprotoporphyrin IX) is toxic [4] and as such, represents a major threat to the viability of the parasite (owing to difficulties regarding the nomenclature of iron protoporphyrin IX, for the purpose of this review haem refers to the Fe(III) form of the porphyrin and is used interchangeably with the abbreviated name, Fe(III)PPIX). While comparatively insoluble in such an acidic aqueous environment, Fe(III)PPIX is soluble in a lipid environment and as such has been invoked in lipid peroxidation and membrane damage [5,6]. In what appears to be a detoxification mechanism, malaria parasites sequester free Fe(III)PPIX into a micro-crystalline form known as haemozoin or malaria pigment [7]. It has been shown that at least 95% of the free haem is detoxified in this manner [1]. Earlier work suggested that only as much as 30% of free haem was detoxified as haemozoin. In this case the authors suggested that the remainder was most likely degraded outside of the food vacuole by glutathione [8].

The formation of haemozoin appears to be a common route of haem disposal in a number of haematophagous organisms; indeed its formation has been observed in species as unrelated to plasmodia as the blood-feeding helminth *Scistosoma mansoni* [9] and the blood-sucking triatomine insect *Rhodnius prolixus* [10]. While *Plasmodium* haemozoin comprises well-defined, micron-sized rectangular crystals, the crystalline material recovered from *S. mansoni* and *R. prolixus* is less homogenous and differs markedly in overall morphology [11]. Despite these differences in external appearance, it has been shown that all haemozoin crystals are isostructural and share the same unit cell. Recently, Pisciotta *et al*. have shown that crystals of *Plasmodium* haemozoin occur within nanospheres predominantly formed of neutral lipids inside the food vacuole [12]. Supported by insights from molecular dynamics simulations, it has been suggested that the lipid/aqueous interface that these bodies present is key to the formation of haemozoin crystals [13]. Similarly, haemozoin formation within *S. mansoni* for example, has been shown to take place in lipid droplets within the gut lumen [11]. Readers are directed to three recent reviews on haemozoin for further details [14,15,16].

The focus of the current review is the recent developments in the fields of HTS and the discovery of new haem-targeting scaffolds via target-based structural design. The examples reviewed typically have involved some aspect of molecular modelling in order to arrive at the final compounds. While the compounds discussed have their intended use as antimalarial agents, they could eventually be of chemotherapeutic value to treat schistosomiasis given a common means of haem disposal. 

## Chemotherapy of Human Malaria and Schistosomiasis

**Figure 1:**
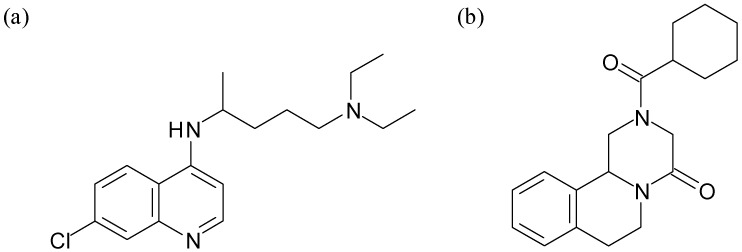
The mainstays of malaria and schistosomiasis chemotherapy: (a) chloroquine and (b) praziquantel, respectively.

Historically, malaria chemotherapy has largely been achieved through the use of quinoline-based antimalarial drugs, of which chloroquine (Figure 1a), a 4-aminoquinoline compound, has enjoyed the greatest success [17]. Amodiaquine, also a 4-aminoquinoline compound, as well as the quinoline methanol drugs quinine and mefloquine, have also been widely used in the treatment of the disease. Related to the quinolines, the phenanthrene methanol drugs halofantrine and lumefantrine have had widely different success rates. On recommendation from the World Health Organisation due to its adverse side effects, halofanrine (Halfan) no longer appears on the medicine formularies of most countries [18,19]. Coartem, a combination of artemether and lumefantrine, has very recently received FDA approval [20] and in one study [21], was shown to be the most effective treatment in areas where resistance to usual treatments is high. Chloroquine is active against the growing ring and trophozoite stages [22] of the parasite (*P. falciparum*) life cycle, during which large quantities of haemoglobin are digested, suggesting that drug treatment may interfere with parasite feeding. Morphologically, chloroquine has been reported to cause vacuolar swelling as well as clumping of haemozoin crystals [23,24], which indirectly supports this notion. It is now generally accepted that 4-aminoquinoline and possibly also quinoline- and phenanthrene-methanol antimalarial compounds target haem (Fe(III)PPIX) to inhibit the formation of haemozoin [25,26,27]. There is an extensive literature demonstrating that 4-aminoquinoline, quinoline- and phenanthrene methanol antimalarials inhibit the abiotic formation of β-haematin, the synthetic equivalent of haemozoin, and that these inhibitory activities correlate with their antimalarial activities [28,29,30,31,32,33,34]. Interestingly, the inactive stereoisomers of quinine, 9-epiquinine [28] and 9-epiquinidine [35], do not inhibit β-haematin formation or do so very weakly.

To date, the drug of choice in the treatment of schistosomiasis has been the compound praziquantel (Figure 1b) [36]. While the drug target remains unknown, investigations suggest that calcium channels may be involved [37,38]. The inhibition of haemozoin formation does not appear to be the target of this successful drug. One study emanating from the group of Oliveira demonstrated that praziquantel, up to concentrations of 1 mmol/dm^3^, did not inhibit the *in vitro* aggregation of haem induced by total female worm homogenates [39]. In the same study, motivated by growing parasitic resistance towards praziquantel as well as the fact that these haematophagous organisms share with *Plasmodium* a common means of haem disposal, the activity of chloroquine against *S. mansoni* was investigated. In an initial *in vitro* study it was found that chloroquine inhibited the aggregation of haem induced by particulate fractions of *S. mansoni* female homogenates. The effect was dose-dependent, with an apparent IC_50_ of 30 µmol/dm^3^. The *in vivo* activity of chloroquine on *S. mansoni* infection in adult Swiss mice was then later investigated according to two distinctly different dosing protocols. In the first, mice were injected daily between days 42 and 49 after infection, during which time adult helminths reportedly undergo a period of intense haemoglobin digestion. In the second protocol, in order to investigate drug activity against the young lung stages as they begin to feed on blood, the mice were injected every other day between days 7 and 28 following infection. While chloroquine did not cause a significant decrease in the overall haemozoin content of adult female *S. mansoni*, it did reduce their viability considerably, most likely due to oxidative stress caused by the small increase in free haem as a result of haemozoin inhibition. Importantly however, the treatment administered in protocol 1 neither reduced the overall parasitemia nor the number of eggs deposited in the host liver. Conversely, the regime followed in protocol 2 resulted in significant decreases in haemozoin inhibition as well as reduced parasitemia of both male and female worms. Most notably, treatment with chloroquine effected a concomitant decrease in the number of eggs that were deposited in the host liver.

The schistosomicidal activity of a second antimalarial drug, mefloquine, has also been investigated [40]. A single 150 mg/kg dosage of racemic mefloquine was delivered to infected male Swiss mice eight weeks post infection in order to determine its effect on the total worm count, egg development and viability and the functioning of intestinal mast cells. In a similar manner to chloroquine, mefloquine treatment did result in decreased numbers of eggs, specifically those in the early stages of development. However, it did not bring about significant decreases in the total worm count. Furthermore, mefloquine alone, as observed in mefloquine-treated, uninfected mice, was shown to induce intestinal mastocytosis, an over-production of mast cells. The resultant symptoms are also associated with schistosomiasis and were therefore heightened in mefloquine-treated infected mice. While the schistosomicidal mechanism of action of mefloquine is not known, the authors suggest that the drug interferes with bloodfluke reproduction as opposed to previous thoughts of it slowing waste excretion. That mefloquine may act via the inhibition of haemozoin formation as has been suggested in the case of malaria infection was not proposed. It is evident that this question still requires much research.

Despite the historical success of chloroquine as an antimalarial, its decline as both a suitable chemotherapeutic agent and a prophylactic is now widespread [41,42]. This is owing to parasitic resistance. Genetic mutations in the genes that encode two membrane transport proteins, namely *P. falciparum* CQ-resistance transporter (*Pf*CRT) and *P. falciparum* P-glycoprotein homologue 1 (*Pf*Pgh1), have been correlated with quinoline resistance [43,44]. Resistance to mefloquine has been associated with the amplification of the gene encoding the latter protein as has cross-resistance to quinine and halofantrine [45]. The emergence of drug-resistant malaria and vulnerability to the potential development of drug resistance in schistosomiasis is the driving force behind the research effort to develop new and effective compounds. Importantly however, in the case of malaria infection at least, the processes of haem metabolism and haemozoin formation remain viable drug targets. It is conceivable therefore, that improved methods of high-throughput screening for haemozoin inhibitors coupled with a better understanding of the mechanism of action of existent haemozoin inhibitors are likely to be of great value in the discovery and rational design of novel compounds.

## Advances in High-Throughput Screening Methods

Following the identification of a suitable drug target, the process of research and development of novel chemotherapeutic agents relies upon the discovery of suitable chemical lead compounds and the optimisation of their activity [46]. Specifically, high-throughput screening (HTS) is a choice method for sifting through thousands of compounds in a relatively short space of time. A survey of the literature reveals that while several laboratories are working towards improved β-haematin inhibition assays that may find usefulness in a HTS context (reviewed in reference [47]), little work has so far been reported in which true HTS has been performed in the sense of screening thousands of unknown compounds. Of particular note, however, is a recently developed assay [48] which has been designed such that it mimics the lipid-rich environment of the digestive vacuole where haemozoin formation most likely takes place [9,12,13]. Reportedly, it has been validated for HTS of large compound libraries. The results of actual HTS of compounds in the search for novel haem-targeting pharmacophores, both experimentally [50,51] and *in silico* [50,52] are focused on here. In the first instance, libraries of drug candidates are assayed for inhibition activity against haemozoin formation. The latter application relies on some prior knowledge of the structural and chemical features which are necessary for activity [53], in essence the pharmacophore of an existent drug.

**Figure 2:**
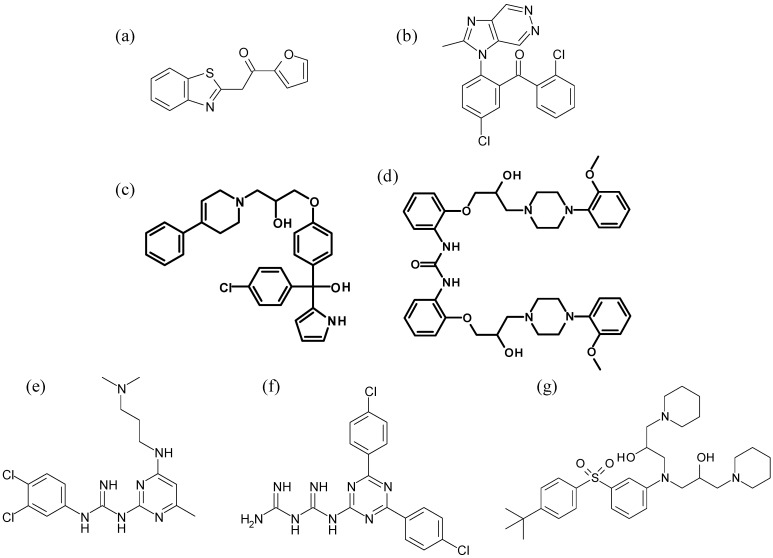
Haemozoin inhibitors identified through HTS [50]. The compound classes (a) miscellaneous, (b) benzophenone, (c) triarylcarbinol and (d) piperazine were identified experimentally, while compounds (c) – (g) were identified using *in silico* methods. The structures in bold [(c) and (d)] represent the intersection of these two methods.

One of the few investigations that exemplifies the strength of the HTS method for discovering novel haemozoin inhibiting antimalarial pharmacophores was described in 2000 [50]. Employing a radioactive ^14^C-haem “polymerisation” assay [54] in which lipid-rich trophozoite lysate extracts in acetonitrile were used to promote haemozoin formation, the strength of inhibition of haemozoin formation of over 100,000 compounds was tested. Haemozoin formation was brought about in 96-well filtration microplates and HTS was semi-automated using a robot. In the search for potential lead compounds, only those which deviated from the quinoline nucleus and which displayed an IC_50_ less than 50 µM were considered further. Of the 45 non-quinolines identified, only a subset of four displayed activities of less than 5 µM against both chloroquine-sensitive and –resistant *P. falciparum* strains (Figure 2). While each compound would require modification towards improved sensitivity before being considered a useful drug candidate, the strength of a random HTS process for the discovery of novel haemozoin inhibitors had been proven. In the same study, an *in silico* screening of the Roche database for compounds with structural features analogous to 4-aminoquinolines was performed using the pharmacophore-generating software Catalyst. Compounds were compared to a “consensus structure” which had been determined on the basis of the three-dimensional structure of each selected compound together with indices ranking its lipophilicity, hydrogen bond donor and acceptor positions and electrostatic relationships. Of the 317 compounds that satisfied the distance constraints of the “consensus structure”, 26 displayed activities in the range 0.15–1.0 µM against *P. falciparum* strains. Five of these were new structures (Figure 2). Notably, the intersection of the subsets identified by HTS and *in silico* methods included two compounds, a triarylcarbinol and a piperazine, demonstrating the strength of a virtual screening process in the identification of novel haem-targeting pharmacophores. 

**Figure 3:**
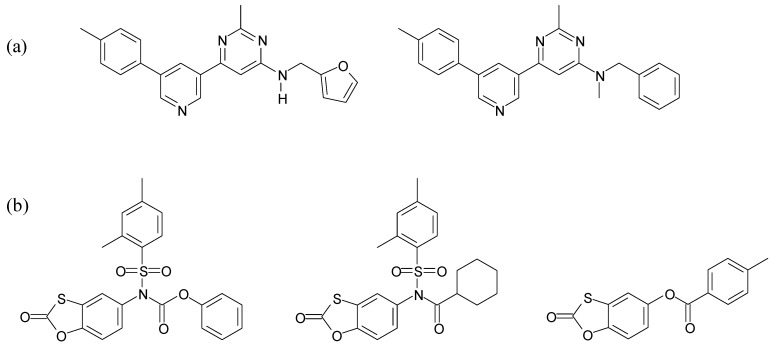
Classes of novel haem-targeting chemotypes identified through a modified HTS assay [51]: (a) pyrimidines and (b) 1,3-benzoxathiol-2-ones.

Very recently, Rush *et al*. [51] described a modified 384-well HTS assay based on the 96-well colorimetric Phiβ (pyridine hemichrome inhibition of β-haematin) assay previously reported [55]. In addition to the increased microtitre plate format, important adaptations include a reduced acetate buffer concentration from 12.9 M to 9.7 M for optimal mechanical handling at room temperature. This necessitates a two-hour incubation time compared to the one hour used previously in order to bring about maximum β-haematin formation. Volumes of reagents and solvents were also varied in order to achieve reproducible data. In its optimised form, this assay has been used to screen more than 16,000 structurally diverse compounds. Emanating from the screen were some 644 compounds (hit rate ~ 4%) with *in vitro* β-haematin inhibitory IC_50_ values of 220 μM or less and reproducibilities between replicates of close to 1. These hits were subsequently assayed for their activity to inhibit *P. falciparum* growth and the two data sets compared *in silico*. 17 compounds (final hit rate ~ 0.1%) were identified for their ability to inhibit resistant parasite strains at IC_50 _concentrations less than 20 μM. Two classes of structurally related compounds were common to the 17 hits and represent novel haem-targeting chemotypes (Figure 3).

While the works of Kurosawa *et al*. [50] and Rush *et al*. [51] demonstrate the usefulness of virtual screening as a means of identifying the effectiveness of different compounds, pharmacophore models have also been applied in an attempt to predict the activities of novel compounds. In one study [52], 250 conformations of a *training* set of 23 previously evaluated molecules (mostly quinolines) [33] were used to generate three pharmacophore models for haem-targeting antimalarials (based on 1) inhibitors of haemozoin formation, 2) those active against chloroquine-sensitive strains and 3) those active against chloroquine-resistant strains). The model based on activity against chloroquine-resistant strains of *P. falciparum* was selected following its success in a Fischer’s randomisation test. Hydrogen bond acceptor ability, aliphatic hydrophobicity and the availability of a protonation site (ionisability) were considered essential chemical features for activity while the presence of an aromatic ring system was deemed necessary but not sufficient for activity. As a check, this model was used to back-predict the activities of the training set. Somewhat disappointingly, halofantrine was the only molecule to satisfy all four chemical features. Several other molecules lacked one or more of the essential features in the correct position. For example, the model predicted that chloroquine’s ionisable feature was placed incorrectly. This is surprising given the success of chloroquine as an antimalarial and its ability to concentrate in its diprotonated form inside the digestive vacuole via pH trapping [14]. Despite incongruities in the fitting of the training set molecules, the pharmacophore model was then used in a predictive capacity to evaluate the activities of a *test* set of 55 previously evaluated molecules which differed substantially in structure from those in the training set. Of interest are the results generated against the 12 highly active (< 50 µM) compounds. Six were predicted correctly, two were predicted as moderately active and four were predicted as inactive. The family to which the latter four compounds belong, the cryptolepines, was not represented in the training set. The authors suggest that their rigid structure resulted in a poor correlation as too few conformations could be attained. In spite of this obvious failing, the authors promote their model for its ability to have correctly predicted the highly active floxacrine derivative WR243246. However, on careful reading of the original publication [56], the active compound is in fact WR243251 while WR243246, the ketone hydrolysis product of the former compound, was shown to be inactive against haemozoin formation (haem “polymerisation”). On this basis, it was further suggested that WR243246 may act according to a completely different mechanism rather than haemozoin formation. Such discrepancies showcase a possible drawback of the virtual screening process: the range of new molecules that may be successfully predicted is really only as good as the data used during the generation of the model. Chloroquine would not have been predicted as highly active had it been in the test set of compounds. 

**Figure 4:**
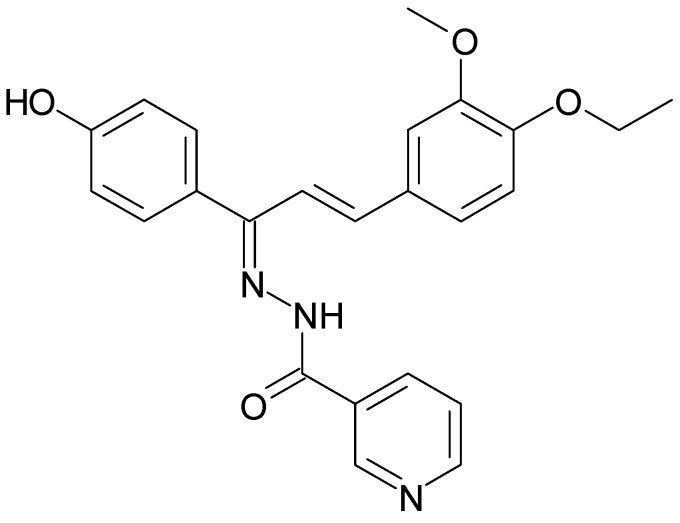
A potentially new haem-targeting antimalarial compound discovered by virtual screening: nicotinic acid [trans-3-(4-ethoxy-3-methoxyphenyl)-1-(4-hydroxyphenyl)-allylidene]-hydrazide [57].

In a more recent publication by the same authors [57], a pharmacophore model for haem-targeting antimalarials was developed on the basis of structurally diverse moleceules, not necessarily quinolines. In keeping with the previous model [52], aliphatic hydrophobicity, ionisability and the presence of an aromatic ring system were identified as important features in order to successfully map the training set molecules. However, hydrogen bond donor ability was preferred to hydrogen bond acceptor ability in the new model. This model was validated against a test set of molecules and was able to correctly predict the activities in 65% of cases. In order to test the model further, an in-house library of 90 compounds was screened *in silico*. A nicotinic acid derivative (Figure 4) was returned which had an estimated activity of 160 nM, making it fall within the moderately active category. As before however, the only feature that was not correctly mapped by the model was the ionisability feature. Taking into account the calculated vacuolar accumulation ratios, the authors report an activity of 129 ± 12 nM for the synthesised compound against the RKL9 chloroquine resistant strain. 

While the works of Acharya and Kaushik [52,57] do showcase a method which provides insight about the active pharmacophore of haem-targeting antimalarials, the question remains how to ensure that potentially valuable novel compounds are not passed over during *in silico* screening of chemical libraries due to incorrect mapping? 

## Interactions of Existing Antimalarials with Haem

The absence of indisputable structural evidence of the interactions of antimalarial drugs with haem has hampered the rational design of novel compounds. To date, such efforts have relied largely on evidence derived from spectroscopic measurements and theoretical calculations and the avid reader is directed to a comprehensive review concerning the interaction of quinoline antimalarials with Fe(III)PPIX in solution for further details [58]. Essentially however, the literature suggests that electronic (π – π) interactions contribute significantly to complex formation. Investigations involving arylmethanol compounds such as quinine and its stereoisomers have further suggested that coordination of the iron centre of Fe(III)PPIX and related porphyrins by the alcohol/alkoxy functionality is possible [59,60,61]. Surprisingly, both of the inactive isomers (9-epiquinine and 9-epiquinidine) associate with Fe(III)PPIX equally or more strongly than quinine [34,35,62]. Such anomalies hint at the importance of definitively determining the structures of the Fe(III)PPIX–alkaloid complexes in order to understand drug activity. However, despite several studies having investigated the interactions of quinoline and phenanthrene methanol compounds with Fe(III)PPIX, the structure-activity relationship for this class of compounds remains poorly understood.

**Figure 5:**
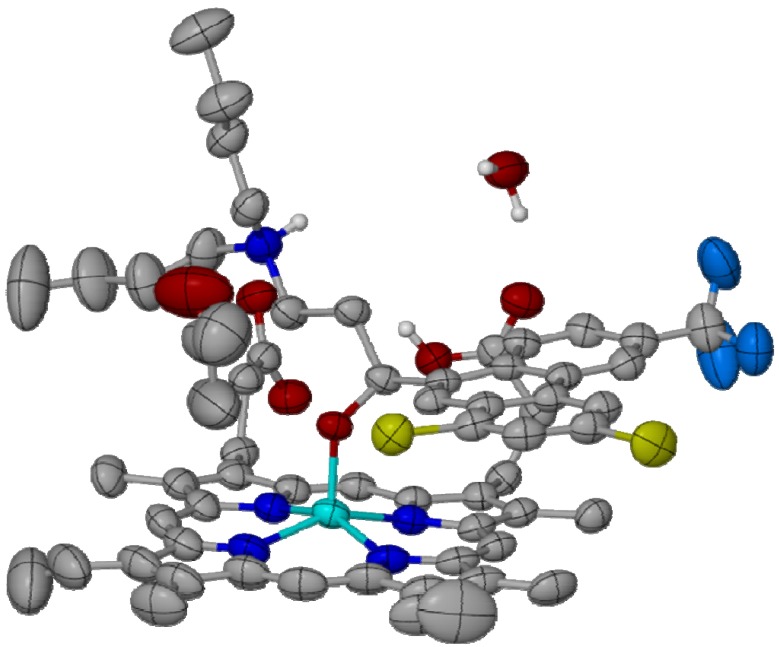
The crystal structure of the Fe(III)PPIX-halofantrine complex. Thermal ellipses are drawn at 50% probability, illustrating significant disorder in the Fe(III)PPIX vinyl groups, butyl chain termini of halofantrine and the included acetone molecule. Atom colouring: C: grey, Cl: yellow; F: Prussian blue, Fe: cyan, H: white, N: blue, O: red. Reprinted with permission from reference [63]. Copyright 2008 Elsevier.

A major development in understanding the interaction of existing antimalarials, specifically the arylmethanol compounds, with Fe(III)PPIX has been the recent elucidation of the crystal structure of its complex with halofantrine [63]. It has been shown that in addition to the envisaged *π*-stacking of the phenanthrene ring of halofantrine over the porphyrin, the drug coordinates to the iron centre of monomeric Fe(III)PPIX via its alcohol functionality (Figure 5). The reported Fe–O bond length is consistent with the coordination of an alkoxide rather than an alcohol. Amongst others, an important *inter*molecular hydrogen bond occurs between the protonated terminal nitrogen atom of halofantrine and the propionate group of a neighbouring Fe(III)PPIX molecule. While this crystal structure provides invaluable insight into the mode of interaction of halofantrine with haem, it is to be remembered that it is a structural representation of the molecular interactions that take place in the solid state. Furthermore, on the basis of a lipid environment having been identified as important for the formation of haemozoin/β-haematin [12], drug inhibition of this process is more than likely to take place in the same non-aqueous, hydrophobic environment [64]. Yet, the experimental conditions from which the Fe(III)PPIX-halofantrine complex was crystallised are largely aqueous. 

Aware of these obvious weak points, the authors [63] undertook a spectroscopic investigation of the association between the free base of halofantrine and Fe(III)PPIX in acetonitrile, a low-dielectric solvent that provides a model of the non-aqueous, hydrophobic environment in which haemozoin inhibition has been inferred to take place. While the structure of the Fe(III)PPIX-halofantrine complex, either in the solid state or in solution, is unable to predict analogous behaviour for antimalarial compounds that do not possess an alcohol functional group, it was deemed likely that it may provide a model for the interaction of other arylmethanol antimalarials with Fe(III)PPIX, most notably quinine and its stereoisomers. Indeed, the authors were able to show that the spectroscopic changes upon titration of an acetonitrile solution of Fe(III)PPIX with the free base form of quinidine were strikingly similar to those observed for halofantrine. A subsequent molecular dynamics/simulated annealing investigation of the coordination complexes formed between quinine and its stereoisomers (quinidine, 9-epiquinine and 9-epiquinidine) and Fe(III)PPIX revealed only minor energy differences in their minimum energy conformations. However, when a dummy *intra*molecular hydrogen bond was included between the protonated quaternary quinuclidine nitrogen atom of a drug molecule and the negatively charged propionate side chain of the Fe(III)PPIX molecule to which it was complexed, the resultant energy penalties (calculated as the difference in energy between the minimum energy and hydrogen bonded conformations in each case) were shown to correlate with the biological activities of the four alkaloid compounds. The authors proposed that the favourable formation of an *intra*molecular hydrogen bond in the case of quinine and quinidine prevents dissociation of the drug molecule from Fe(III)PPIX, thus essentially blocking the iron centre from coordination by a second molecule of Fe(III)PPIX as proposed in a recent model of haemozoin dimer formation [13].

On the basis of these findings, the authors put forward a novel pharmacophore for haem-targeting compounds. Their model consists of a three-point interaction that involves π-stacking, coordination and intramolecular hydrogen bonding. It remains to be seen if this model will lend to rationally-designed novel haemozoin inhibitors with antimalarial activity.

**Figure 6:**
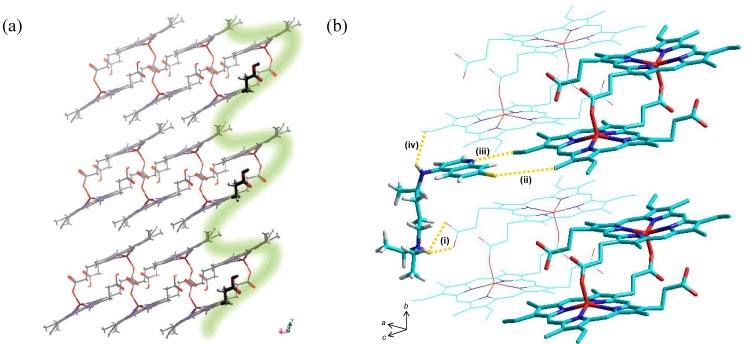
(a) A model of β-haematin viewed along the *a* axis. The corrugation of the (001) face is indicated by the green line. Reprinted with permission from reference [65]. Copyright 2002 American Chemical Society. Quinolines (for example chloroquine) are able to dock into the grooves and are stabilised by various close contacts discussed in the text (b). The original coordinates for β-haematin were used to build the crystal [67].

By way of an alternative, haemozoin itself has been suggested as a possible target for antimalarial drugs [65,66]. Leiserowitz and co-workers hypothesised that antimalarial compounds are able to adsorb onto the surface of haemozoin crystals, specifically to the fastest-growing (001) and (00-1) faces [65]. These faces are highly corrugated, exposing flexible propionic acid groups, vinyl and methyl groups, as well as aromatic surfaces in grooves which run parallel to the *a* axis (Figure 6a). Thus drug adsorption is stabilised by close contacts with these surrounding functional groups. Using monoprotonated chloroquine (CQ) as one example, the authors identified the following possible noncovalent interactions (Figure 6b): (i) a 2.7 Å (porphyrin) COO^-^····(CQ) R_3_NH^+^ salt bridge, (ii) a 3.0 Å (porphyrin) CH_3_····(CQ) Cl contact, (iii) a 2.4 Å (porphyrin) C=CH····(CQ) N_quinoline_ contact and (iv) a 2.7 Å (porphyrin) C=C(π cloud)····(CQ) NH_4-amino_ contact. In addition, the planar quinoline ring is able to intercalate between planar porphyrin rings in this model. One possible criticism of the model is that quinolines were used in a monoprotonated state, making possible the (porphyrin) C=CH····(CQ) N_quinoline_ interaction. As discussed previously, chloroquine is expected to accumulate in the digestive food vacuole as a diprotic weak base. The work does however represent an additional, and yet to be explored, pharmacophore model for the rational design of haem-targeting drugs. 

## Drug Discovery: Pharmacophore-Based Drug Design

One approach towards producing a new drug compound involves the chemical modification of existing compounds in a particular class [46]. However, owing to a common molecular skeleton and therefore similar structure-activity relationships, drugs discovered in this way may encounter resistance very quickly. A second approach relies on the rational design of novel compounds based on a sound understanding of both parasite biology and the chosen molecular target [46]. It is not to say that the second approach can stand apart from chemical synthesis; indeed, once formulated, the next hurdle is to be able to prepare novel compounds via relatively simple and low-cost synthetic routes. This latter approach is beginning to take root for haem-targeting drugs, and while the literature is not yet replete with examples, some recent advances in the rational discovery of novel antimalarials of this type are discussed here.

**Figure 7:**
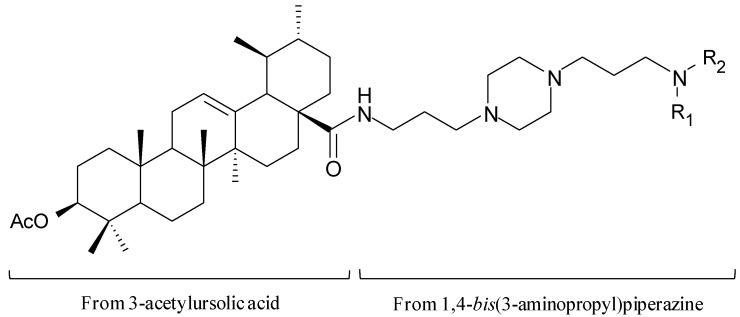
Piperazinyl derivatives of acetylursolic acid with nanomolar *in vitro* activities against FcB1 *P. falciparum* [68]. (a) R_1_ = R_2_ = H, (b) the deacetylated version of (a), (c) R_1_ = R_2_ = 4-hydroxybenzyl, (d) R_1_ = R_2_ = methylcyclopropyl, (e) R_1_ = R_2_ = propyl, (f) R_1_ = H, R_2_ = 4-hydroxybenzyl, (g) R_1_ = H, R_2_ = methylferrocenyl.

In early 2008, Gnoatto *et al*. reported the pharmacomodulation of a series of novel antimalarial compounds synthesised via the condensation of the saponin derivative 3-acetylursolic acid and 1,4-*bis*(3-aminopropyl)piperazine [68]. Saponins are a class of naturally-occuring amphipathic compounds comprising both hydrophilic glycoside and hydrophobic triterpene moieties. Ursolic acid, a non-aromatic polycyclic hydrocarbon, occurs either as a free acid or as an aglycone of triterpene saponins and the antimalarial activities of combinatorial libraries based on its non-π-stacking scaffold alone have been investigated previously [69]. The inclusion of the 1,4-*bis*(3-aminopropyl)piperazine moiety was based upon a previous study which suggested that a salt bridge formed between the protonated piperazine nitrogen atom and the carboxylate side chain of haem may aid antimalarial activity [70]. A series of 21 new compounds were synthesised according to the abovementioned design strategy and tested for both β-haematin inhibitory activity and *in vitro* sensitivity against chloroquine-resistant and -sensitive *P. falciparum* strains (FcB1 and Thai respectively) [68]. Seven new piperazinyl compounds (Figure 7) were found to exhibit significant (80–800 nM) *in vitro* activity against FcB1 compared to chloroquine (130 nM). The resistance indices of these compounds (in the range 0.37–1.17) indicate that their level of cross-resistance to chloroquine is low. Two of the most active compounds overall (Figure 7, a and c) demonstrated good *in vitro* inhibition of β-haematin as well, suggesting a possible common mechanism of action with chloroquine. One downfall is that all novel compounds emanating from this study were observed to be cytotoxic in a mammalian cell line (MRC-5). The authors suggest however, that with a selectivity index of 27, the compound shown in Figure 7f may be a good lead for further development.

**Figure 8:**
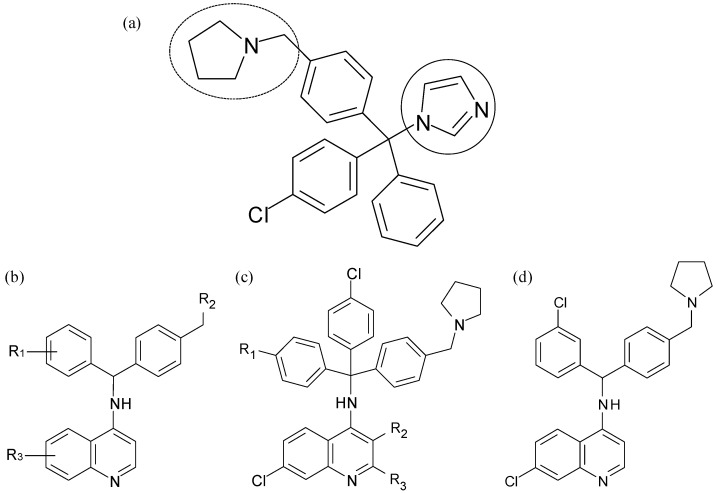
Novel clotrimazole-based antimalarial compounds. (a) A typical clotrimazole derivative showing imidazole (solid ring) and protonatable amine (dashed ring) [73]. Generalised hybrid compounds combining a 4-aminoquinoline nucleus with (b) a simplified benzhydryl moiety or (c) the triarylmethyl moiety of clotrimazole. (d) The 4-aminoquinoline-benzhydryl analogue chosen for preclinical studies.

Exploiting the malaria parasite’s own biochemistry and its specific need to maintain tightly-controlled redox and antioxidant systems inside the red blood cell [71], Gemma *et al*. followed a similar hybridisation strategy to that above [72]. Their rationale was based on previous work on a series of clotrimazole-based molecules in which an imidazole ring is directly bonded via its nitrogen atom to a polyarylmethyl moiety [73]. The hypothesis followed that the imidazole group may be able to coordinate to the iron centre of Fe(II)PPIX and in so doing, induce a “conjugation-mediated electron transfer reaction” that would result in the formation of a trityl radical species within the digestive food vacuole of the malaria parasite. A protonatable amine functionality positioned along a lateral chain was also found to enhance the activity of the clotrimazole-based molecules, owing to improved accumulation in the digestive food vacuole (Figure 8a). Thus, through the combination of the well-known 4-aminoquinoline nucleus (in place of the imidazole group in the above example) with the same polyarylmethyl moiety as above, the authors were able to synthesise a series of novel hybrid antimalarials [72]. Axial coordination of the iron centre of haem was proposed via the quinoline nitrogen atom in order to generate the toxic radical intermediates. While several of the novel hybrid compounds showed potent activity against chloroquine-resistant *P. falciparum* strains, one in particular (Figure 8d) proved an excellent lead compound. It displayed good activity even after oral administration, a promising pharmacokinetic profile, no antimycotic activity and very low cytotoxity and was thus processed further for preclinical studies.

The work of Riscoe *et al*. involving xanthone derivatives has also lead to the discovery of a novel haem-targeting scaffold [74]. These two-ring aromatic systems were later modified to three in order to improve their ability to interact with haem through π-stacking. The resultant tricyclic molecules exhibit low nanomolar *in vitro* activities against *P. falciparum* and were further shown to inhibit haemozoin formation. The acridone nucleus, also a tricyclic aromatic system, was preferred for its nitrogen atom at the 10-position, which would later become the location for further substitution. Of the unsubstituted derivatives however, the most potent were the haloalkoxyacridones; they exhibited *in vitro* activities down to the picomolar level and were not cytotoxic even at concentrations 100,000 times greater [75]. 

**Figure 9:**
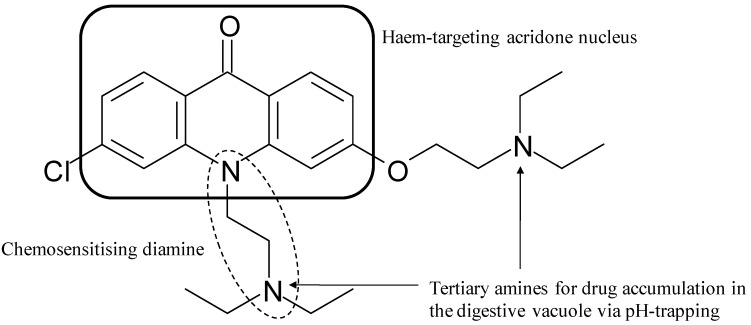
Acridone derivative T3.5, highlighting the haem-targeting nucleus overlapping the chemosensitising diamine moiety. Redrawn based on the original reference [77].

This work laid the foundation for the rational design of a third example of novel antimalarials based on the combination of known pharmacophores [76,77]. In a unique manner, the 10-N position on the planar haem-targeting acridone pharmacophore was exploited to attach a known [78] chemosensitising (resistance-reversing) pharmacophore. The resultant compounds were, for the first time, a combination of overlapping pharmacophores as opposed to being separated by a linker or formulated in a single dosage as two individual components. The initial lead compound (T3.5, Figure 9) has shown both *in vitro* and *in vivo* activities against chloroquine-sensitive and multi-drug resistant *P. falciparum* strains that are comparable to quinine and chloroquine. Furthermore, the authors demonstrated synergy between T3.5 and the quinoline drugs chloroquine, quinine, amodiaquine and piperaquine in support of these compounds aiding the efficacy of current antimalarials. 

## Conclusions

Significant advances in our understanding of the mechanism by which haemozoin occurs have taken place in the last two to three years. Notions that the formation of this crystalline material is an autocatalytic or protein-mediated process have been largely dispelled in light of compelling evidence that natural haemozoin exists enveloped inside neutral lipid bodies within the digestive vacuole of *P. falciparum*. Coupled with the favourable fact that the process of haemozoin formation is unique to the parasite, this mechanistic insight renders the pathway of haem disposal an invaluable target for rational drug design. This is supported by the large number of registered antimalarial compounds which have been shown to be effective against haemozoin formation. While optimised HTS assays will no doubt lead to the discovery of novel haem-targeting drugs based on the sheer numbers of compounds tested, the process remains costly due to the large compound databases that need to be sourced, purchased or synthesised. Furthermore, the screening process, at least in the virtual case, will only ever be as good as its design allows, which means that some potentially excellent hits may be passed over if their structural information is not embedded in the hypothesis. This review has highlighted examples of work in which novel antimalarial scaffolds have been discovered following a rational design approach and it is noteworthy that several of the reported lead compounds emanating from these studies exhibit activities that are comparable to or better than chloroquine, the historical mainstay of malaria chemotherapy. These early success stories are likely to accelerate malaria drug discovery initiatives and given that the target process of haem disposal is common to other blood-feeding organisms, it could be of great benefit should lead compounds discovered in one program be of value to another. Malaria and schistosomiasis account for a large proportion of global disease. It is not inconceivable that a multilateral effort towards drug development may result in a drug that is active against multiple diseases. However, time will tell.
